# Frizzled-7 is required for *Xenopus* heart development

**DOI:** 10.1242/bio.026963

**Published:** 2017-11-03

**Authors:** Muhammad Abu-Elmagd, Joanna Mulvaney, Grant N. Wheeler

**Affiliations:** 1 Center of Excellence in Genomic Medicine Research, King Abdulaziz University, P.O. Box 80216 Jeddah 21589, Kingdom of Saudi Arabia; 2 School of Biological Sciences, University of East Anglia, Norwich Research Park, Norwich, NR4 7TJ, UK

**Keywords:** *Xenopus laevis*, Cardiogenesis, Wnt signalling, Fzd7

## Abstract

Wnt signalling regulates cardiogenesis during specification of heart tissue and the morphogenetic movements necessary to form the linear heart. Wnt11-mediated non-canonical signalling promotes early cardiac development whilst Wnt11-R, which is expressed later, also signals through the non-canonical pathway to promote heart development. It is unclear which Frizzled proteins mediate these interactions. Frizzled-7 (*fzd7*) is expressed during gastrulation in the mesodermal cells fated to become heart, and then in the primary heart field. This expression is complementary to the expression of *wnt11* and *wnt11-R*. We further show co-localisation of *fzd7* with other early- and late-heart-specific markers using double *in situ* hybridisation. We have used loss of function analysis to determine the role of *fzd7* during heart development. Morpholino antisense oligonucleotide-mediated knockdown of Fzd7 results in effects on heart development, similar to that caused by Wnt11 loss of function. Surprisingly, overexpression of dominant-negative Fzd7 cysteine rich domain (Fzd7 CRD) results in a cardia bifida phenotype, similar to the loss of *wnt11-R* phenotype. Overexpression of Fzd7 and activation of non-canonical wnt signalling can rescue the effect of Fzd7 CRD. We propose that Fzd7 has an important role during *Xenopus* heart development.

## Abstract (precis)

**Summary:** Wnt signalling has been shown to be important in heart development. Here, we demonstrate that the wnt receptor *fzd7* is required in mediating these Wnt signals.

## INTRODUCTION

During embryogenesis, the heart is one of the first organs to form. Development of the heart includes specification of cardiac progenitors and formation of the linear heart tube by cell migration and morphogenetic movements (Mohun et al., 2000). In *Xenopus*, the heart begins to form during early gastrula stages when the cardiac progenitors arise in the dorsolateral mesoderm. Cell movements during gastrulation result in the dorso-anterior translocation of these regions and subsequent ventral migration during neurulation. The heart progenitors, which comprise cells fated to become primary or secondary heart field, form a linear heart tube at the ventral midline before looping and remodelling to form the beating heart (Kriegmair et al., 2013). Understanding the processes underlying heart development and morphogenesis are important for understanding congenital heart disease.

Heart formation is controlled by many signalling pathways including wnt signalling. *Wnt6*, *wnt11*, and *wnt11-R* have all been implicated in *Xenopus* heart development (Garriock et al., 2005; Gessert et al., 2008; Lavery et al., 2008a; Pandur et al., 2002). Wnt antagonists such as Dickkopf-1, Crescent and Sfrp1 have also been reported to control early heart formation (David et al., 2008; Foley and Mercola, 2005; Gibb et al., 2013; Marvin et al., 2001; Schneider and Mercola, 2001). Little is known however about which Frizzled proteins mediate these signals. Frizzled-7 (*fzd7*) has been well characterised in *Xenopus laevis* and other species. It has been shown to be involved in numerous developmental processes as well as being shown to be active in several forms of cancer (Huang and Klein, 2004; Liu et al., 2016; Schiffgens et al., 2016; Xu et al., 2016). *Fzd7* has been demonstrated to interact with several wnts including Wnt5a (animal cap elongation assays), Wnt6 (in somite development), Wnt8 (co-immunoprecipitation assays, *Xenopus* axis duplication) and Wnt11 (gastrulation movements, neural crest development) (Hsieh et al., 1999; Linker et al., 2005; Medina et al., 2000; Medina and Steinbeisser, 2000; Umbhauer et al., 2000; Witzel et al., 2006). It has also been shown to genetically interact with the co-receptors *ror2* and *ryk* (Hikasa et al., 2002; Kim et al., 2008). *Xenopus fzd7* has been implicated in gastrulation movements, tissue separation, and neural crest induction (Abu-Elmagd et al., 2006; Djiane et al., 2000; Wheeler et al., 2000; Winklbauer et al., 2001). We have previously shown *fzd7* to be expressed in the cardiac region throughout development (Wheeler and Hoppler, 1999). It has also been shown that specific depletion of *fzd7* function in *Xenopus* foregut leads to impaired cardiac morphogenesis, but has no effect on heart specification (Zhang et al., 2013). Here, we further characterise its expression relative to known heart markers, and then use whole-embryo experiments to show that *fzd7* is required for heart formation during early embryonic development.

## RESULTS

### *fzd7* expression overlaps with early heart markers

**Fig. 1.**
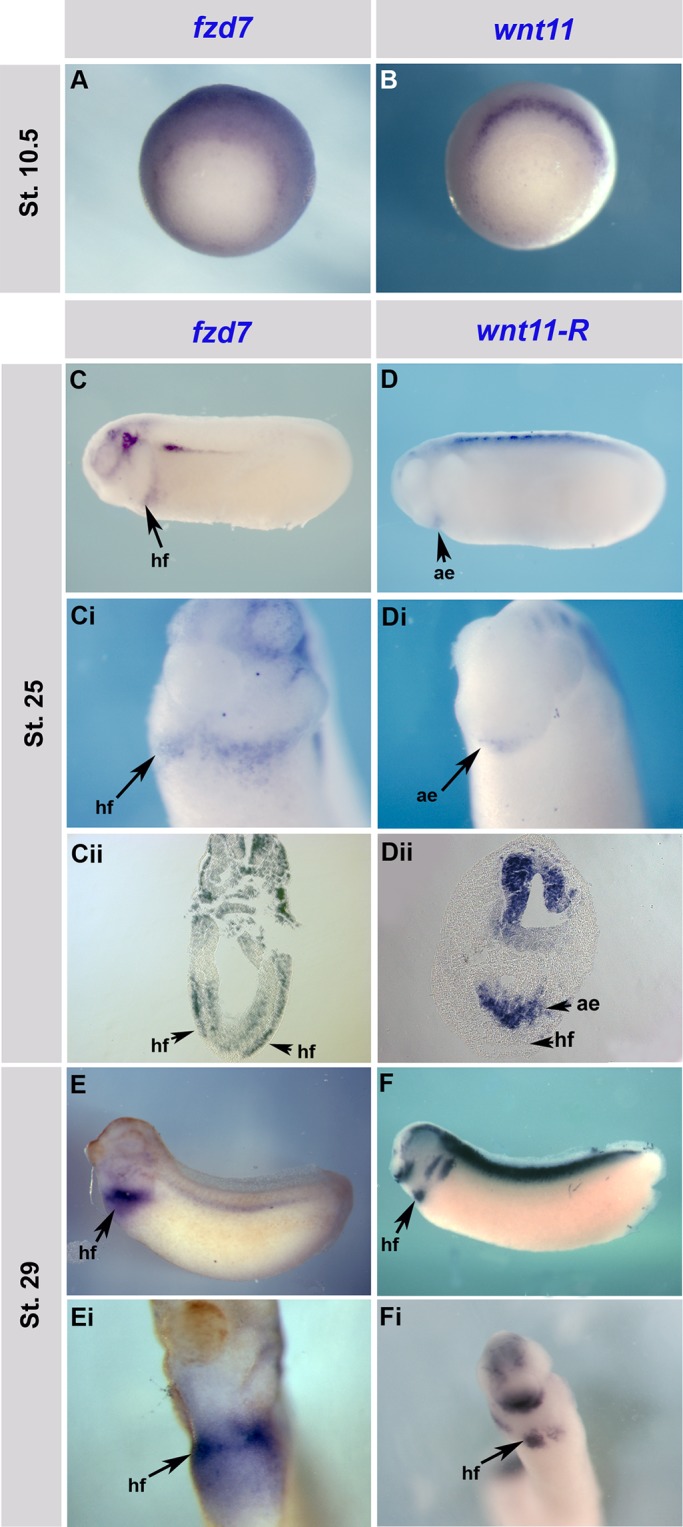
**Endogenous expression of *fzd7* in *Xenopus* heart and relative to *wnt11* and *wnt11-R* expression.** (A,B) Stage 10.5 (mid-gastrula) *fzd7* and *wnt11* expression detected at the dorsal side of the embryo and appear complementary in the presumptive heart region. (C-Cii,D-Dii) *fzd7* and *wnt11-R* expression at stage 25. *fzd7* is seen in the heart field and *wnt11-R* in the anterior endoderm. *fzd7* and *wnt11-R* expression are complementary in the heart region (Cii,Dii). (E,Ei,F,Fi) Stage 29 embryos with *fzd7* and *wnt11-R* expression in the heart field. hf, heart field; ae, anterior endoderm. Magnification 20×.

**Fig. 2.**
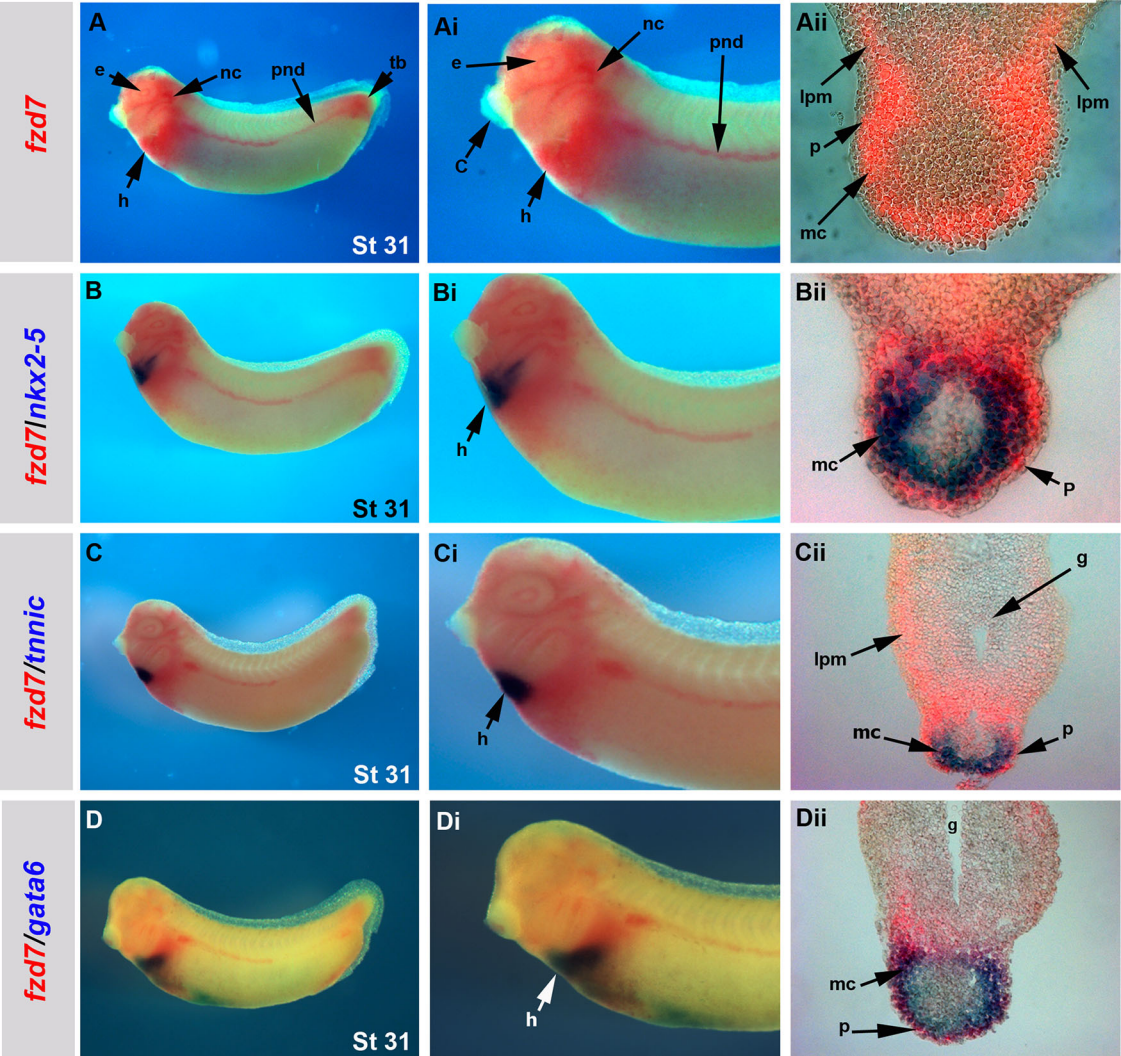
***fzd7* expression coincides with expression of the early heart markers *nkx2-5*, *tnnic* and *gata6*.** (A-Aii) Lateral view of *Xenopus laevis* embryos at stage 31 showing *fzd7* expression detected in red and co-localised by double *in situ* hybridisation with other heart markers in dark blue including *nkx2-5* (B-Bii), *tnnic* (C-Cii) and *gata6* (D-Dii). (Ai,Bi,Ci,Di) Magnified lateral view of the same embryos in A, B, C and D, respectively. (Aii,Bii,Cii,Dii) Cross sections through the heart region of the embryos in A, B, C and D, respectively. *fzd7* is expressed in the myocardium and pericardium (Aii) and in other structures including neural crest, eye, pronephric duct and tail bud. *fzd7* expression shows a high degree of overlapping with the heart markers in the myocardium but not in the pericardium (Bii,Cii,Dii). h, heart; c, cement gland; e, eye; nc, neural crest; pnd, pronephric duct; tb, tail bud; mc, myodcardium; lpm, lateral plate of mesoderm. Magnification: 20× in A, B, C and D; 30× in Ai, Bi, Ci and Di; 200× in Aii, Bii, Cii and Dii.

Expression pattern analysis shows *Xenopus fzd7* is expressed in the heart-forming regions throughout development (Wheeler and Hoppler, 1999). At stage 10.5 *fzd7* is expressed in the dorsal mesoderm from which cardiac tissue originates (Wheeler and Hoppler, 1999) (Fig. 1A). As development progresses, *fzd7* expression at stage 25 is maintained in the presumptive cardiac mesoderm as it migrates dorso-laterally to the ventral midline (Fig. 1C-Cii). By stage 29, *fzd7* is expressed throughout the cardiac crescent in the cardiac mesoderm (Fig. 1E,Ei). *fzd7* expression correlates with that of *wnt11* (Fig. 1B, stage 10.5) where expression of both genes seem to be complementary in the presumptive heart region in the dorsal side of the embryo. *fzd7* expression also correlates to that of *wnt11-R* (Fig. 1D-Dii,F,Fi, stages 25 and 29, respectively) where it is expressed in the anterior endoderm at stage 25 when *fzd7* is expressed in the heart field. By stage 29, the expression of *fzd7* and *wnt11-R* overlaps (Fig. 1E-Fi). As the heart continues to form, *fzd7* is strongly expressed in the lateral plates of mesoderm, cardiac mesoderm, myocardium, and over time, is restricted to the pericardium (Wheeler and Hoppler, 1999) (Fig. 2A-Aii, Bii, Cii, Dii). Using double *in situ* hybridisation, we analysed *fzd7* expression in correlation to that of early heart markers including *nkx2-5*, *troponin-ic* (*tnnic*) and *gata6*, which are all known to be required for *Xenopus* cardiogenesis (Afouda and Hoppler, 2011; Afouda et al., 2008; Drysdale et al., 1994; Flaherty and Dawn, 2008; Fu et al., 1998; Garriock et al., 2005; Jiang and Evans, 1996; Martin et al., 2010). *fzd7* expression overlaps with that of *nkx2-5* (Fig. 2B-Bii), *tnnic* (Fig. 2C-Cii) and *gata6* (Fig. 2D-Dii) in the forming heart. Interestingly, none of these markers are seen in the pericardium except for *fzd7* (Fig. 2Aii,Bii,Cii,Dii).

### *fzd7* is required for heart induction or specification

Microinjection into *Xenopus* embryo dorsal blastomeres at the 4- or 8-cell stage targets prospective mesoderm including cardiac tissue. In order to test the role of *fzd7* in heart development, we inhibited its function by injecting either *fzd7* morpholino (*fzd7* MO) or its dominant-negative form expressing only the extracellular domain (cysteine rich domain, *fzd7* CRD), which would disrupt *fzd7* mediated signalling (Abu-Elmagd et al., 2006).

**Fig. 3.**
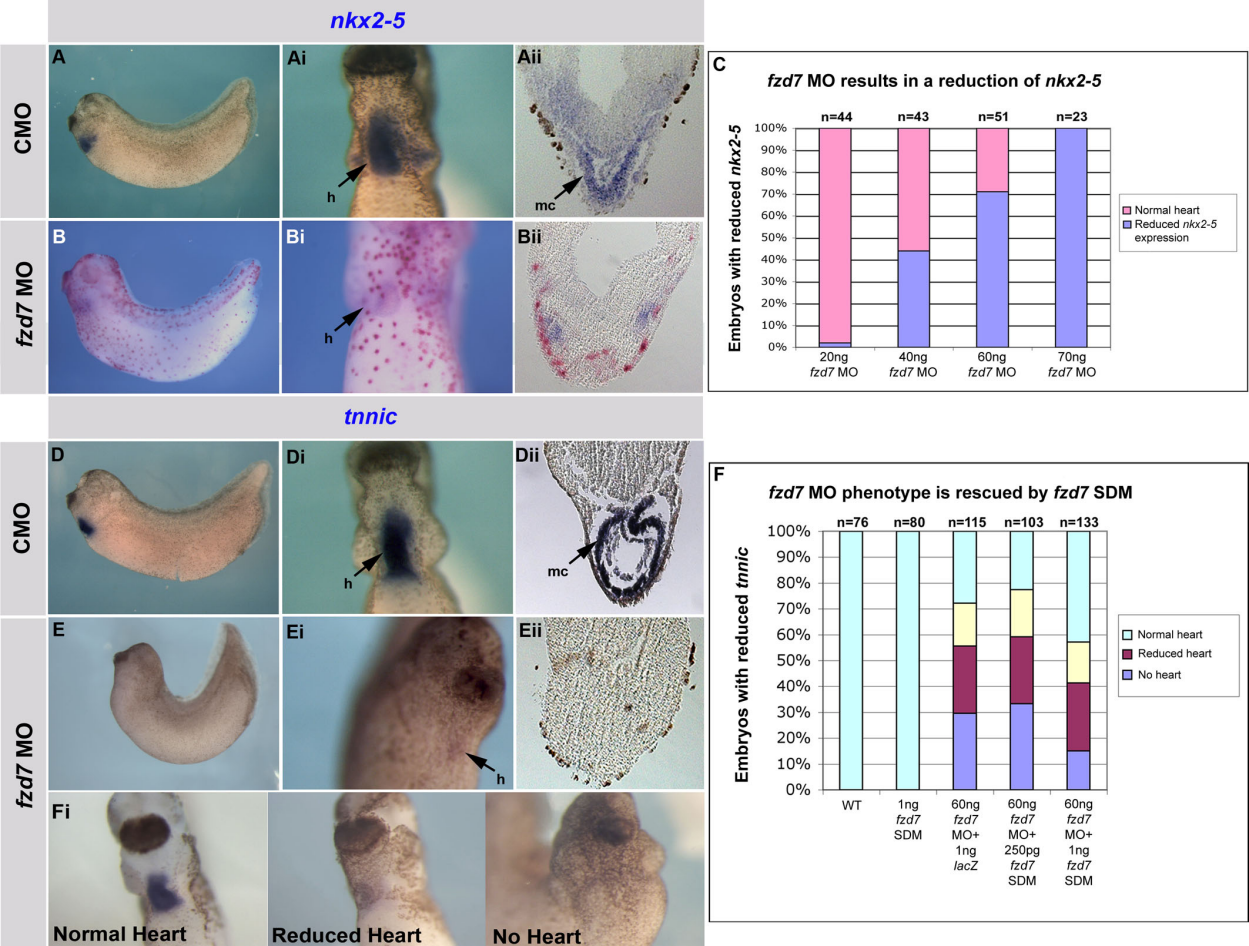
***fzd7* is required for *Xenopus* heart development.** (A,Ai,D,Di) Lateral and ventral views of embryos injected in the dorsal blastomeres (DB) at 4-cell stage with control morpholino (CMO) showing normal *nkx2-5* (A,Ai) and *tnnic* (D,Di) expression. (Aii,Dii) Cross sections in the heart region of the embryos in A and D, respectively, showing normal *nkx2-5* and *tnnic* expression in the myocardium. (B,Bi,E,Ei) Lateral and ventral views of embryos injected in the DB at 4-cell stage with *fzd7* MO showing loss of *nkx2-5* (B-Bi) and *tnnic* (E,Ei) expression. (Bii,Eii) Cross sections in the heart region of the embryos in B and E, respectively, showing loss of the heart. (C) Graph showing that *fzd7* MO phenotype leads to reduction of *nkx2-5* expression in a dose-dependent manner. (F,Fi) *fzd7* MO phenotype can be rescued by *fzd7* SDM full-length, panel Fi is the key for the phenotype scoring. Red staining in B-Bii is due to *lac-Z* lineage tracing using Red-Gal.

Microinjection of *fzd7* MO into the dorsal blastomeres of 4- or 8-cell embryos leads to a reduction of both early cardiac marker *nkx2-5* (Fig. 3B-Bii) and later cardiac marker *tnnic* expression (Fig. 3E-Eii). Adding increasing amounts of *fzd7* MO leads to a progressively more severe phenotype with a greater number of embryos affected (Fig. 3C). *In situ* hybridisation for *nkx2-5* and *tnnic* show embryos with mild convergent extension phenotypes (Fig. 3B,E), but a severe decrease in cardiac gene expression (Fig. 3Bi,Ei) while control morpholino (CMO) show normal heart (Fig. 3A,Ai,D,Di). Some embryos also showed anterior defects (data not shown). Sections through the cardiac region showed not only a decrease of *nkx2-5* and *tnnic* expression, but an absence of recognisable heart structures (Fig. 3Bii,Eii) compared to CMO (Fig. 3Aii,Dii). The number of embryos injected with *fzd7* MO which showed heart and/or convergent extension and anterior defects are shown in Table S1.

Overexpression of *fzd7* full-length (*fzd7* FL) results in severe convergent extension defects, but no cardiac phenotype (Fig. S1A,B). Knockdown with *fzd7* MO can also cause a mild convergent extension phenotype and anterior defects (Abu-Elmagd et al., 2006). In order to test whether this cardiac effect is specific to *fzd7*, we rescued the *fzd7* MO cardiac phenotype with *fzd7* FL that has been mutated to not bind the *fzd7* MO (*fzd7* SDM, as described in Abu-Elmagd et al., 2006). Titrating increasing amounts of *fzd7* SDM capped RNA from 250 pg to 1 ng results in a modest rescue of the cardiac phenotype (Fig. 3F,Fi; Table S2), thus showing that *fzd7* is required for normal heart development.

Interestingly, injecting *fzd7* FL at 8-cell stage embryos shows detectable expression of *tnnic* and *nkx2-5*, despite some of these embryos showing severe convergent extension movements phenotype (head arrows in Fig. S1A,B). This leads to the suggestion that heart phenotypes are not necessarily due to convergent extension secondary effects.

### *fzd7* CRD mimics *wnt11-R* morpholino cardia bifida phenotype and is required for non-canonical signalling

**Fig. 4.**
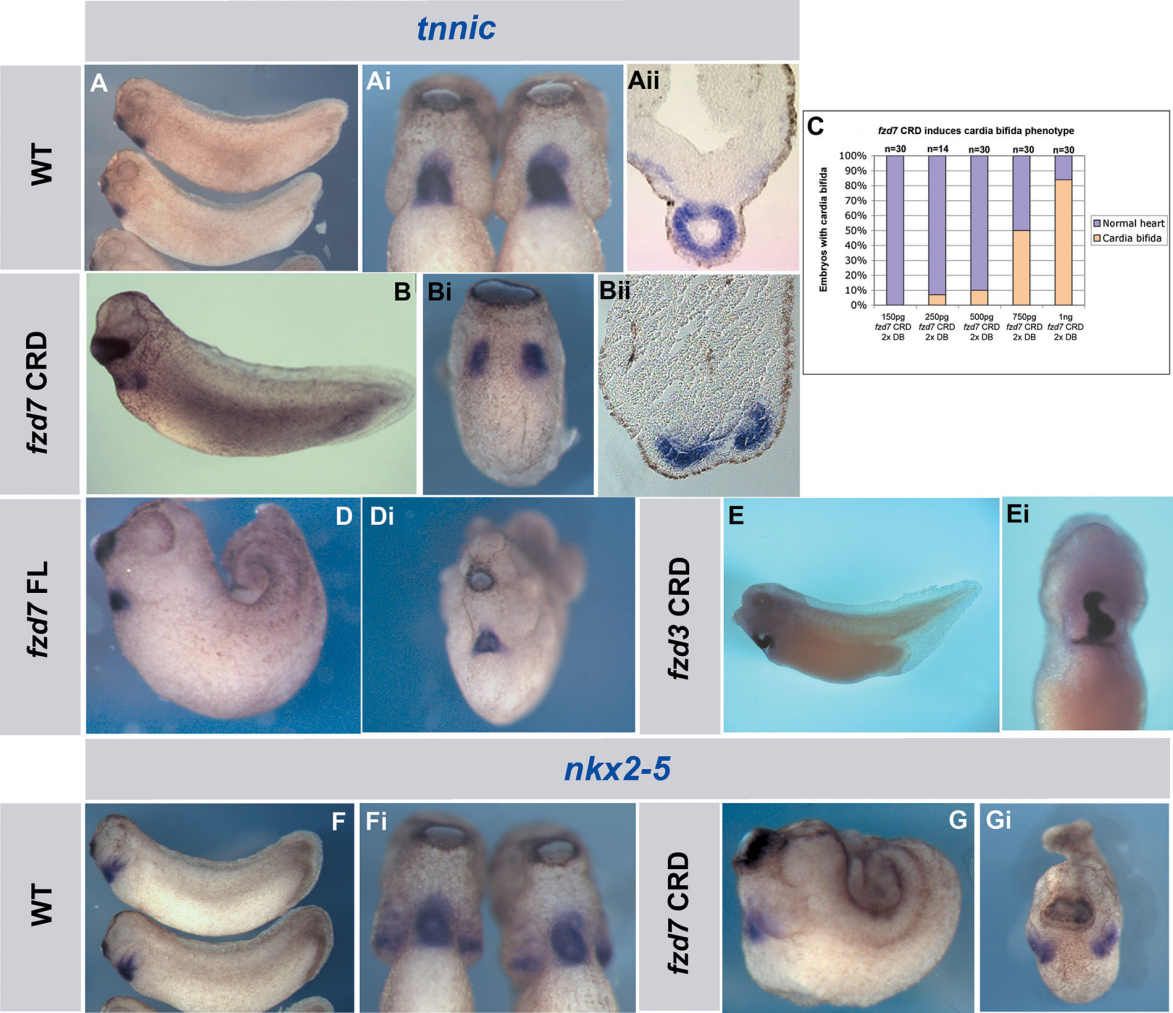
**A dominant-negative *fzd7* induces cardia bifida phenotype.** (A,Ai,F,Fi) Lateral and ventral views of wild-type embryos at stage 29 showing normal *tnnic* (A, Ai) and *nkx2-5* (F-Fi) expression in the heart. (B,Bi,G,Gi) Lateral and ventral views of embryos injected in the DB at 4-cell stage with dominant-negative *fzd7* (*fzd7* CRD). The cardia bifida phenotype is shown by *tnnic* (B,Bi) and *nkx2-5* (G,Gi) expression. These embryos were fixed at the same stage as the control embryos in A and F. (C) Graph showing *fzd7* CRD cardia bifida phenotype percentages indicated by *tnnic* expression. (D,Di) Lateral and ventral views of embryos injected in the DB at 4-cell stage with full-length of *fzd7* (*fzd7* FL) showing normal heart tube. Note that embryos in D and G are showing severe convergent extension defects but cardia bifida phenotype is only induced by *fzd7* CRD. (E,Ei) Lateral and ventral views of injected embryo in the DB at 4-cell stage with *fzd3* dominant-negative form (*fzd3* CRD) showing normal heart looping at stage 38 indicating that *fzd7* CRD cardia bifida phenotype is specific to *fzd7*. Magnification 20×.

**Fig. 5.**
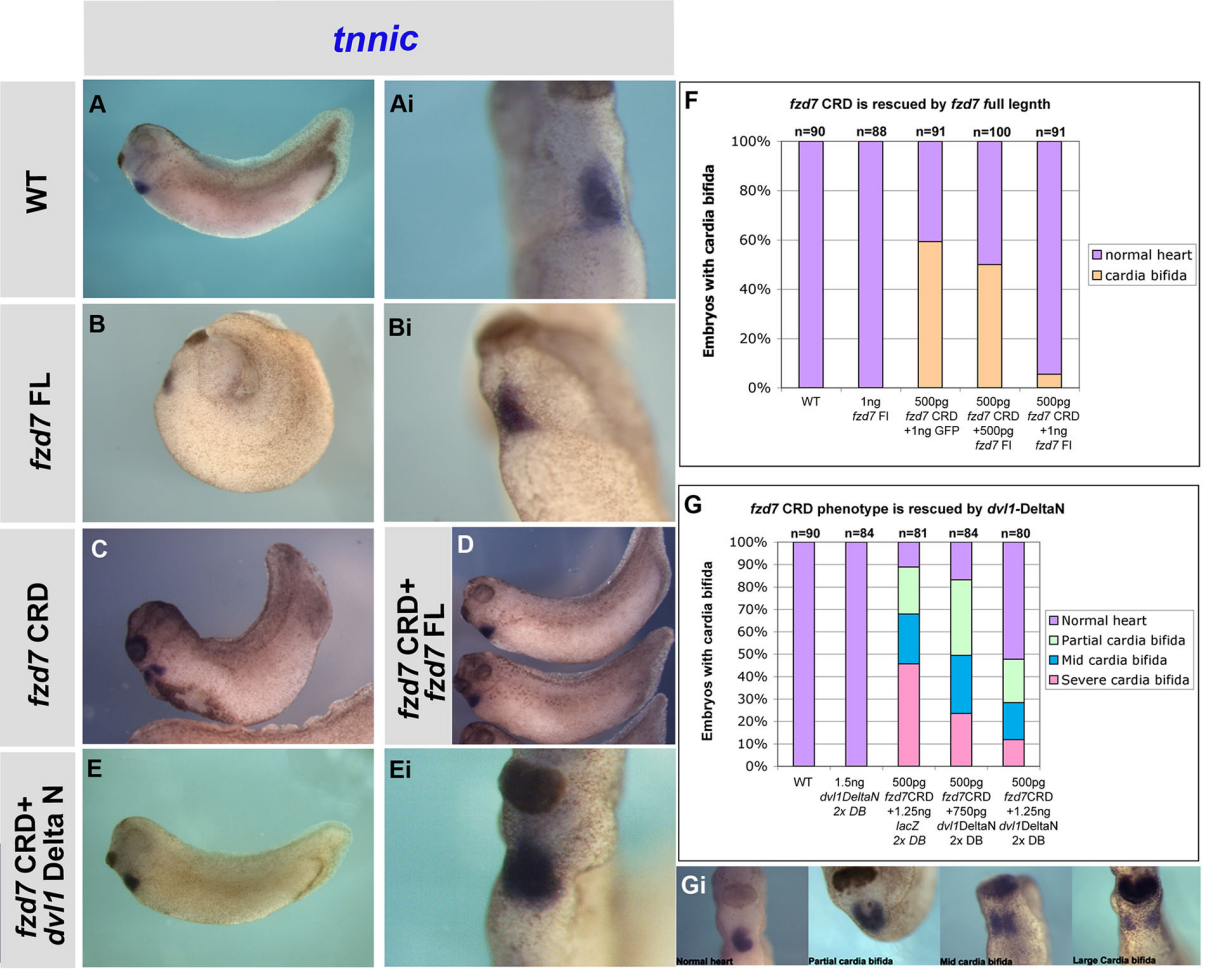
**Activation of non-canonical wnt signalling rescues *fzd7* CRD-induced cardia bifida.** (A,Ai) Wild-type control embryos showing normal *tnnic* expression in the heart. (B,Bi) *fzd7* full-length (*fzd7* FL) overexpression (500 pg) injected into the dorsal blastomeres (DB) at the 4-cell stage show normal heart expression of *tnnic* despite suffering a severe extension movement defect. (C) Embryos injected with 500 pg *fzd7* CRD show cardia bifida phenotype, note that embryos have normal to mild convergent extension defects. (D) Rescue of the *fzd7* CRD (250 pg) cardia bifida phenotype with 250 pg *fzd7* FL, embryos show normal morphology as well as normal *tnnic* expression. (F) Graph of *fzd7* CRD cardia bifida phenotype rescue with *fzd7* FL. (E,Ei) Rescue of *fzd7* CRD (500 pg) cardia bifida phenotype with *dishevelled1*-Delta-N (Dvl1ΔN, 1.25 ng) indicating that *fzd7* is required for the non-canonical signalling in the heart. (G) Graph of *fzd7* CRD cardia bifida phenotype rescue with *dvl1*ΔN, panel Gi is the key for the cardia bifida phenotype scoring in G. Magnification 20×.

To further look at the effect of inhibiting *fzd7* function, we took a dominant-negative approach using *fzd7* CRD. Surprisingly, this did not give a similar result to the MO knockdown. Instead, increasing amounts of *fzd7* CRD results in a dose-dependent increase in frequency and severity of cardia bifida. This was very similar to the phenotype seen for *wnt-11R* knockdown (Garriock et al., 2005). Embryos with very mild convergent extension movement defects displayed a severe cardia bifida phenotype as shown by *tnnic* (Fig. 4B-Bii,C) and *nkx2-5* (Fig. 4G,Gi) expression. Control embryos showed normal expression of *tnnic* (Fig. 4A-Aii) and *nkx2-5* (Fig. 4F,Fi). These results suggest that the cardia bifida phenotype is not a secondary effect of the convergent extension defect. Overexpression of *fzd7* FL gives a severe convergent extension phenotype but no cardiac phenotype (Fig. S1A,B). Embryos with cardia bifida were unable to recover and form a normal heart when incubated up to stage 41 (*n*=23, data not shown). Embryos injected with a dominant-negative form of *fzd3* (*fzd3* CRD) into the dorsal blastomeres at 4-cell stage did not show cardia bifida (*n*= 27, Fig. 4E,Ei) indicating that the cardia bifida phenotype is specific to *fzd7* CRD. Furthermore, this phenotypic specificity to *fzd7* CRD was confirmed by rescuing the cardia bifida with *fzd7* FL-capped RNA (Fig. 5A-D,F).

It has been previously reported that a Jun N-terminal kinases (Jun) inhibitor phenocopies the *wnt11-R* cardiac phenotype of effects on cardiac morphogenesis and heart tube fusion, suggesting signalling through the non-canonical pathway (Garriock et al., 2005; Gessert et al., 2008). We therefore determined to rescue the *fzd7* CRD phenotype with dishevelled1-Delta-N (*dvl1*ΔN)-capped RNA. *Dvl1*ΔN-capped RNA can rescue *fzd7* CRD (Fig. 5E,Ei,G,Gi; Table S3), suggesting that *fzd7* is required for non-canonical wnt signalling during heart development.

## DISCUSSION

Wnt signalling through the canonical and non-canonical pathways has been implicated in many aspects of heart development (Gessert and Kuhl, 2010; Ruiz-Villalba et al., 2016). How the wnt signals that arise from both non-cardiogenic and cardiogenic tissue are integrated into heart development is less well understood. Frizzled proteins are only a part of the increasingly complicated wnt-receptor complex found at the cell membrane, which can also include Lrp5/6, Ror2, Ryk and Kremen (Bryja et al., 2009; Korol et al., 2008; Mazzotta et al., 2016; van Wijk et al., 2009); however, Frizzled proteins are critical components of the Wnt receptor complex and so understanding their role in heart development is necessary to fully understand the signalling involved. We have previously shown that *fzd7* is expressed throughout heart development, and in this study, we show that it is functionally required in both early and late heart development.

Morpholino knockdown of *fzd7* leads to effects on heart development, including in some cases a complete loss of heart (Fig. 3). Overexpression of *fzd7* gives rise to convergent extension defects as previously reported (Abu-Elmagd et al., 2006; Sumanas and Ekker, 2001; Winklbauer et al., 2001), but does not affect heart development. We can rescue the *fzd7* MO phenotype by co-injecting site-directed mutagenized full-length *fzd7* (Fig. 3). These results suggest that *fzd7* is required for initial heart development, though we cannot exclude the possibility that it may also be playing a more general role in dorsoventral mesoderm patterning. Fzd7 could be interacting with Wnt11 (Kim et al., 2008; Tao et al., 2005; Witzel et al., 2006) , or another wnt ligand such as Wnt3a (Mazzotta et al., 2016), Wnt6 (Gibb et al., 2013; Lavery et al., 2008a, b) or Wnt8c (Ruiz-Villalba et al., 2016; Schneider and Mercola, 2001) during these stages of development.

As suggested, it is possible that the *fzd7* morphant cardiac phenotype is a secondary effect of failures in mesoderm specification, patterning, gastrulation, axis formation and tissue separation. We have made efforts to inject embryos at the 4- and 8-cell stages to give as small a convergent extension phenotype as possible to generate normal-looking embryos but with clear heart phenotypes. The results suggest that the effect of *fzd7* during early heart development is not secondary to convergent extension defects or mesoderm development, however, this cannot be ruled out completely (Fig. 3).

An interesting feature of the loss-of-function analysis using *fzd7* Morpholino and a dominant-negative *fzd7* (*fzd7* CRD), is that they give different cardiac phenotypes. *fzd7* morphants have anterior defects, convergent extension defects and reduction in *nkx2-5* expression; whereas *fzd7* CRD-capped RNA injections result in embryos with convergent extension defects and cardia bifida, but no head defects or loss of cardiac markers. Interestingly, it has been shown that the only way to replicate the anterior defect phenotype with a *fzd7* CRD construct is to inject the capped RNA into oocytes (Medina et al., 2000). This could be because the relevant signalling event has been completed by time the product of mRNA injected at the 4- or 8-cell stage has been generated. It is possible that if we injected oocytes with *fzd7* CRD then we might find embryos showing loss of the heart. Another possibility is that the Morpholino is able to disrupt all Wnt signalling through *fzd7* by preventing translation of Fzd7 protein, but *fzd7* CRD only disrupts non-canonical signalling in this context. The requirement for co-receptors in canonical signalling may allow the CRD to interact with endogenous *fzd7* and any Lrps present allowing the receptor complex aggregates to form. In addition to this, it has been shown to be possible to activate canonical Wnt signalling using CRD constructs (Carron et al., 2003). Perhaps canonical Wnt signalling mediated by *fzd7* early on during development is allowed to proceed by the Fzd7 CRD, but then when *fzd7* switches to mediate non-canonical signalling, the CRD starts to behave as a dominant-negative. Other possibilities are that the Morpholino may have a broader specificity than thought or that the injected RNA of the *fzd7* CRD construct may not be very stable, and thus only provide a short term effect compared to the Morpholino. These possibilities remain to be tested further.

The *fzd7* CRD phenotype is very similar to the *wnt11-R* Morpholino phenotype (Garriock et al., 2005). It has previously been shown that *DM-GRASP/alcam* expression lies downstream of *wnt11-R* signalling and that *DM-GRASP/alcam* can mediate non-canonical wnt signalling effects on morphogenetic movements involved in the developing heart. The *DM-GRASP/alcam* Morpholino phenotype is also similar to the *fzd7* CRD phenotype in that they both lead to a cardia bifida-like phenotype and a thickening of the myocardium. This suggests *fzd7* could be mediating the *wnt11-R* control of *DM-GRASP/alcam* expression. This needs to be investigated further.

Ruiz-Villalba et al. (2016) suggest a model where periodic switching between proliferation and differentiation within the developing heart is mediated by the periodic and reciprocal activity of the canonical and non-canonical wnt pathways. *fzd7* could be playing a crucial role in this process depending upon the Wnts and other receptors expressed at specific times.

In conclusion, we have shown *fzd7* to be involved in heart development. Further investigation is required to determine the specific wnt(s) it is interacting with at different stages of heart development.

## MATERIALS AND METHODS

### Embryo manipulation

All experiments were performed in compliance with the relevant laws and institutional guidelines at the University of East Anglia. The research was approved by the local ethical review committee according to UK Home Office regulations. *Xenopus laevis* embryos were obtained as previously described (Harrison et al., 2004). Staging of the embryos was carried out according to the normal timetable of Nieuwkoop and Faber (Nieuwkoop and Faber, 1994). Embryos at the required stages were fixed in MEMFA, washed in PBS, dehydrated in ascending grades of Methanol/PBS, then stored in 100% MeOH at –20°C until processing for single or double *in situ* hybridisation.

### Constructs

*fzd7* full-length (*fzd7* FL) and dominant-negative form *fzd7*-cysteine rich domain (*fzd7* CRD) were sub-cloned into pCS2+ at *Cla1*–*Xho1* restriction sites as described in Wheeler et al. (2000). *fzd7* MO titration by RNA in the rescue experiments was avoided by creating a site-directed mutagenesis construct of the full-coding sequence of *fzd7* (*fzd7* SDM) as described in Abu-Elmagd et al. (2006). *fzd3* full-length (*fzd3* FL) and *fzd3* CRD were kind gifts from Peter Klein (University of Pennsylvania). Dishevelled construct (*Dvl1-Delta-N*) was a gift from Roberto Mayor (University College, London) (De Calisto et al., 2005).

### *In vitro* capped mRNA synthesis and embryo microinjections

All capped mRNAs of all genes used for RNA injections were prepared according to the manufacturer's instructions using the SP6 mMessage mMachine Ambion kit (Invitrogen™ AM1340). Anti-sense oligonucleotides, morpholinos (MOs), were obtained and designed by Gene Tools (www.gene-tools.com, Oregon, USA) using the reported sequence for the control morpholino (CMO) (5′-CCTCTTACCTCAgTTACAATTTATA-3′) and *fzd7*MO (5′-GCGGAGTGAGCAGAAATCGGCTGA-3′) (Sumanas and Ekker, 2001). MOs were diluted, prepared before use according to the manufacturer's instructions and tested using the *in vitro* translation assay (TNT coupled reticulocyte lysate system, Promega-L4600). For targeting the heart, the DB of the 4- and 8-cell stage embryos were injected as previously described (Lavery et al., 2008a). Capped mRNA and MOs were co-injected with *lac-Z* for lineage tracing. Each experiment was carried out as an internally controlled group. Each experiment was carried out three times and the number of embryos in each class were pooled.

### RNA probe synthesis and *in situ* hybridisation

*fzd7* in pBluescript was linearised with *XbaI* and transcribed by T7; *nkx2-5* was linearised with *BamH1* and transcribed with T7; *troponin-lC* (*tnnic*) was linearised with *XhoI* and transcribed with T3; *gata6* was linearised with *XbaI* and transcribed with T7. Promega probe synthesis manufacturing instructions were followed with *fzd7* probe labelled with Fluorescene-substituted nucleotide (Fl-UTP) and for other heart makers labelled with DIG-substituted nucleotide. Each RNA probe was added to 10 ml hybridisation buffer and stored at –20˚C for *in situ* hybridisation. Single (Harland, 1991) or double (Knecht et al., 1995) *in situ* hybridisation was carried out as previously described (Abu-Elmagd et al., 2006). Anti-Fluorescein was detected using Fast Red tablets (Kelloff et al., 2006) while anti-Digoxigenin was detected with NBT/BCIP. Frozen and wax sectioning were carried out as described (Harrison et al., 2004; Hatch et al., 2016). Images were taken using Leica microscope and Axiovision software.

## Supplementary Material

Supplementary information
