# Supplementary material for: Frizzled-7 is required for Xenopus heart development
Source: Biol Open. 2017 Nov 3;6(12):1861–8. doi: 10.1242/bio.026963 (PMC5769647; doi:10.1242/bio.026963)
Supplement: Supplementary information [file biolopen-6-026963-s1.pdf]

## Supplemental Tables and Figures

### Table S1:

| *fzd7* MO Injections | Dose (ng) | Total No. of Embryos | Anterior Defects | Reduced Heart | Anterior Defects and reduced Heart | Normal Morphology and reduced Heart | % Anterior Defects | % Reduced Heart | % Anterior Defects and reduced Heart | % Normal Morphology and reduced Heart |
|---|---|---|---|---|---|---|---|---|---|---|
| **DB** | 20 | 40 | 8 | 1 | 1 | 0 | 20 | 3 | 3 | 0 |
| **VB** | 20 | 26 | 2 | 0 | 0 | 0 | 8 | 0 | 0 | 0 |
| **DB** | 40 | 43 | 15 | 19 | 15 | 4 | 35 | 44 | 35 | 9 |
| **VB** | 40 | 47 | 3 | 0 | 0 | 0 | 6 | 0 | 0 | 0 |
| **DB** | 60 | 51 | 49 | 36 | 34 | 2 | 96 | 71 | 67 | 4 |
| **VB** | 60 | 66 | 0 | 0 | 0 | 0 | 0 | 0 | 0 | 0 |
| **DB** | 70 | 23 | 23 | 23 | 23 | 0 | 100 | 100 | 100 | 0 |
| **VB** | 70 | 30 | 5 | 0 | 0 | 0 | 17 | 0 | 0 | 0 |

***fzd7* Morpholino dose response.** Increasing amounts of *fzd7* MO were injected at the 4 cell stage into both blastomeres of the dorsal side (DB) of the embryo and ventral side (VB) as a control. Observed phenotypes included a range of convergent extension phenotypes from severe to mild, varying degrees of anterior defects and a reduction of *nkx2-5* or *tnnic* expression.

Biology Open • Supplementary information

## Table S2:

| Embryo injections | Total No. of Embryos | No Heart | Reduced Heart | Normal Heart | % No Heart | % Reduced Heart | % Normal Heart |
|---|---|---|---|---|---|---|---|
| **Non-injected control** | 76 | 0 | 0 | 76 | 0 | 0 | 100 |
| **1ng *fzd7* SDM 2x DB** | 80 | 0 | 0 | 80 | 0 | 0 | 100 |
| **1ng *fzd7* SDM 2x VB** | 79 | 0 | 0 | 79 | 0 | 0 | 100 |
| **60ng *fzd7* MO 2x DB** | 52 | 26 | 15 | 11 | 50 | 29 | 21 |
| **60ng *fzd7* MO 2x VB** | 50 | 1 | 3 | 46 | 2 | 6 | 92 |
| **60ng *fzd7* MO + 250pg *lacZ* 2x DB** | 93 | 31 | 41 | 21 | 33 | 44 | 23 |
| **60ng *fzd7* MO + 250pg *lacZ* 2x VB** | 79 | 0 | 0 | 79 | 0 | 0 | 100 |
| **60ng *fzd7* MO + 500pg *lacZ* 2x DB** | 101 | 40 | 33 | 28 | 40 | 32 | 28 |
| **60ng *fzd7* MO + 500pg *lacZ* 2x VB** | 89 | 3 | 0 | 86 | 3 | 0 | 97 |
| **60ng *fzd7* MO + 750pg *lacZ* 2x DB** | 101 | 27 | 53 | 21 | 27 | 52 | 21 |
| **60ng *fzd7* MO + 750pg *lacZ* 2x VB** | 74 | 0 | 0 | 74 | 0 | 0 | 100 |
| **60ng *fzd7* MO + 1ng *lacZ* 2x DB** | 115 | 34 | 49 | 32 | 29 | 43 | 28 |
| **60ng *fzd7* MO + 1ng *lacZ* 2x VB** | 75 | 0 | 1 | 74 | 0 | 1 | 99 |
| **60ng *fzd7* MO + 250pg *fzd7* SDM 2x DB** | 52 | 17 | 26 | 9 | 33 | 50 | 17 |
| **60ng *fzd7* MO + 250pg *fzd7* SDM 2x VB** | 53 | 0 | 0 | 53 | 0 | 0 | 100 |
| **60ng *fzd7* MO + 500pg *fzd7* SDM 2x DB** | 107 | 16 | 42 | 49 | 15 | 39 | 46 |
| **60ng *fzd7* MO + 500pg *fzd7* SDM 2x VB** | 59 | 0 | 1 | 58 | 0 | 2 | 98 |
| **60ng *fzd7* MO + 750pg *fzd7* SDM 2x DB** | 100 | 14 | 40 | 46 | 14 | 40 | 46 |
| **60ng *fzd7* MO + 750pg *fzd7* SDM 2x VB** | 75 | 0 | 1 | 74 | 0 | 1 | 99 |
| **60ng *fzd7* MO + 1ng *fzd7* SDM 2x DB** | 133 | 20 | 56 | 57 | 15 | 42 | 43 |
| **60ng *fzd7* MO + 1ng *fzd7* SDM 2x VB** | 107 | 0 | 1 | 106 | 0 | 1 | 99 |

***fzd7* MO phenotype is rescued by *fzd7* SDM**. Injecting 1ng of *fzd7*SDM capped RNA does not give a cardiac phenotype. Coinjecting 60 ng *fzd7* MO with from 250pg –1ng of *lacZ* capped RNA gives between 51% and 30% embryos with no heart and between 22% and 29% embryos with normal hearts. Coinjecting with *fzd7* SDM capped RNA from 250pg- 1ng gives a dose responsive decrease of embryos with no heart 33% at 250pg to 15% at 1 ng and an increase in embryos with a normal heart from 18% at 250pg to 43% at 1ng. DB: dorsal blastomeres, VB: ventral blastomeres.

Biology Open • Supplementary information

## Table S3:

| Embryo injections | Total No. of Embryos | Severe Cardia Bifida | Mid. Cardia Bifida | Partial Cardia Bifida | Normal Heart | % Severe Cardia Bifida | % Mid. Cardia Bifida | % Partial Cardia Bifida | % Normal Heart |
|---|---|---|---|---|---|---|---|---|---|
| **Non-injected control** | 90 | 0 | 0 | 0 | 90 | 0 | 0 | 0 | 100 |
| **1.5ng *dvl1*ΔN 2x DB** | 84 | 0 | 0 | 0 | 84 | 0 | 0 | 0 | 100 |
| **1.5ng *dvl1* ΔN 2x VB** | 88 | 0 | 0 | 0 | 88 | 0 | 0 | 0 | 100 |
| **500pg *fzd7* CRD + 750pg *lacZ* 2x DB** | 92 | 29 | 25 | 23 | 15 | 32 | 27 | 25 | 16 |
| **500pg *fzd7* CRD + 750pg *lacZ* 2x VB** | 75 | 0 | 0 | 0 | 75 | 0 | 0 | 0 | 100 |
| **500pg *fzd7* CRD + 1ng *lacZ* 2x DB** | 67 | 25 | 10 | 22 | 10 | 37 | 15 | 33 | 15 |
| **500pg *fzd7* CRD + 1ng *lacZ* 2x VB** | 62 | 0 | 0 | 0 | 62 | 0 | 0 | 0 | 100 |
| **500pg *fzd7* CRD + 1.25ng *lacZ* 2x DB** | 81 | 37 | 18 | 17 | 9 | 46 | 22 | 21 | 11 |
| **500pg *fzd7* CRD + 1.25ng *lacZ* 2x VB** | 84 | 0 | 0 | 0 | 84 | 0 | 0 | 0 | 100 |
| **500pg *fzd7* CRD + 1.5ng *lacZ* 2x DB** | 37 | 10 | 16 | 8 | 3 | 27 | 43 | 22 | 8 |
| **500pg *fzd7* CRD + 1.5ng *lacZ* 2x VB** | 28 | 0 | 0 | 0 | 28 | 0 | 0 | 0 | 100 |
| **500pg *fzd7* CRD + 750pg *dvl1*ΔN 2x DB** | 89 | 21 | 23 | 30 | 15 | 24 | 26 | 33 | 17 |
| **500pg *fzd7* CRD + 750pg *dvl1* ΔN 2x VB** | 76 | 0 | 0 | 0 | 76 | 0 | 0 | 0 | 100 |
| **500pg *fzd7* CRD + 1ng *dvl1*ΔN 2x DB** | 91 | 6 | 24 | 34 | 27 | 7 | 26 | 37 | 30 |
| **500pg *fzd7* CRD + 1ng *dvl1*ΔN 2x VB** | 93 | 0 | 0 | 0 | 93 | 0 | 0 | 0 | 100 |
| **500pg *fzd7* CRD + 1.25ng *dvl1*ΔN 2x DB** | 67 | 8 | 11 | 13 | 35 | 12 | 16 | 19 | 52 |
| **500pg *fzd7* CRD + 1.25ng *dvl1*ΔN 2x VB** | 67 | 0 | 0 | 0 | 67 | 0 | 0 | 0 | 100 |
| **500pg *fzd7* CRD + 1.5 ng *dvl1*ΔN 2x DB** | 20 | 2 | 4 | 10 | 4 | 10 | 20 | 50 | 20 |
| **500pg *fzd7* CRD + 1.5ng *dvl1*ΔN 2x VB** | 30 | 0 | 0 | 0 | 30 | 0 | 0 | 0 | 100 |

***fzd7* CRD is rescued by *dvl1* Δ N.** Injecting 1.5 ng of *dvl1*ΔN capped RNA does not give a cardiac phenotype. Coinjecting 160 ng *fzd7* CRD with from 750pg –1.5ng of *lacZ* capped RNA gives between 33% and 46% embryos with severe cardia bifida and between 17% and 10% embryos with normal hearts. Coinjecting with *dvl1*ΔN capped RNA from 750pg- 1.5ng gives a dose responsive decrease of embryos with severe cardia bifida 25% at 750pg to 7% at 1.25ng and an increase in embryos with a normal heart from 18% at 750pg to 44% at 1.25ng. DB: dorsal blastomeres, VB: ventral blastomeres.

Biology Open • Supplementary information

**Figure S1:**

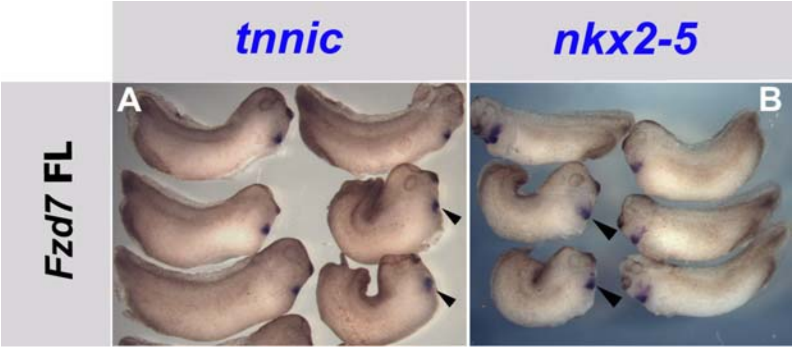


**Cardiac development is independent on the convergent extension movement defects caused by overexpression of *fzd7*.** (A, B). *fzd7* full length (250pg) injected into the dorsal blastomeres at 8 cell stage and incubated till stage-32 showing detectable *tnnic* (A) and *nkx2-5* (B) expression in both normal embryos and those with convergent extension movement defects (arrow heads in A and B).
